# Different Reaction Patterns of Caregivers of Children With Imperforate Anus: A Latent Profile Analysis

**DOI:** 10.3389/fped.2021.796725

**Published:** 2022-02-03

**Authors:** Dan Wang, Hongzhen Xu, Kexian Liu, Jinfa Tou, Yushuang Jia, Wei Gao, Xiaofei Chen, Feixiang Luo

**Affiliations:** ^1^Children's Hospital, School of Medicine, Zhejiang University, Hangzhou, China; ^2^Affiliated Hospital and Yuying Children's Hospital, Wenzhou Medical University, Wenzhou, China; ^3^Anhui Provincial Children's Hospital, Hefei, China

**Keywords:** imperforate anus, caregiver, caregiving reaction pattern, latent profile analysis, cross-sectional study

## Abstract

**Aim:**

This study aimed to explore how different dimensions of caregivers' reaction shape their caring experience, and the factors associated with different reaction patterns.

**Design:**

A second analysis of a multisite cross-sectional study were conducted. Caregivers of children with imperforate anus (IA) were enrolled in three tertiary children's hospitals in Eastern China between November 2018 and February 2019.

**Methods:**

The caregiver's experience, stigma feeling, social support level and perception of uncertainty were assessed by Caregiver Reaction Assessment, Parent Stigma Scale, Social Support Scale and Parent's Perception of Uncertainty Scale accordingly. The demographic information of caregivers as well as the children's clinical data were collected. Latent profile analysis was conducted to determine different patterns of caregiver's reaction, and logistics analysis was used to explore the associated factors of the reaction pattern.

**Findings:**

A total number of 229 caregivers (median age = 30, quartiles: 28, 36) were included. Three distinguishable caregiving reaction types were identified (Class 1: low burden and high benefit, 4.8%; Class 2: moderate burden and benefit, 48.9%; Class 3: high burden and low benefit, 46.3%). In logistics analysis, the Class 1 and Class 2 were combined as one group due to the low population in Class 1. The marital status of caregiver (OR = 0.067, 95% CI: 0.006, 0.700, *P* = 0.024), IA type (OR = 1.745, 95% CI: 1.198, 2.541, *P* = 0.004), children aged > 2 years (OR = 3.219, 95% CI: 1.364, 7.597, *P* = 0.008), social support (OR = 0.907, 95% CI: 0.865, 0.951, *P* < 0.001) and perception of uncertainty (OR = 1.054, 95% CI: 1.026, 1.083, *P* < 0.001) were associated with different caregiver reaction patterns.

**Conclusion:**

Nearly half of the caregivers of children with IA experience reaction of high burden and low benefit, but considerable proportion of caregivers could benefit from the caregiving rather than burden from. Married caregivers may have more negative reaction, especially when children > 2 years and diagnosed with intermediate or high type of IA. However, increasing caregiver's social support and reducing perception of uncertainty may have the potential to modify their reaction pattern.

## Introduction

Imperforate anus (IA) is one of the most common type of anorectal malformation, which is defined as newborn baby without normal opening in the anus area, or only fistula remained (1). With an incidence of 1/5000-1/2000 (2), and difficulties in prenatal diagnosis (3), IA has a higher incidence in developing countries (4), laying high medical burden on national health care.

IA could be divide into three categories according to Wingspread classification, namely low, intermediate and high according to height of the deformity (5). Although Krickenbeck classification has been widely adopted in western countries due to the careful consideration of the anatomical and prognosis features (6, 7), the Wingspread classification remained the mainstream that been used by most children's hospital in developing countries like China (8). Children diagnosed with IA usually need to receive at least one operation (anoplasty). The higher the deformity location is, the more difficulty the treatment is (9). For the intermediate and high type of IA, the three-staged operation usually adopted, including a colostomy, anoplasty and colostomy closure (10). After anoplasty, a long period of dilation is needed to improve the prognosis (11). Despite the advances in operation technology, complications like constipation and incontinence are still common to see in a large number of affected children, as high as 90% children need bowel management (2).

Caring for children with IA could put a lot of burden on the family members, especially the caregivers, who take the most caring tasks. A study showed that, the caregivers were usually under greater pressure than the affected children, and in high risk of getting mental disorder (12). Nearly half of caregivers of children with IA reported impaired life quality (13). However, in despite of the negative feelings from caring for a child with IA, some caregivers could also benefit from the experience of caregiving, and get a sense of self-worth (12, 14). The various feelings related to caregiving could be called caregiver reaction (15).

Caregiver reaction is the perception of the caregiving experience, and caregiver's feelings toward caregiving could be affected by diverse factors. Negatively speaking, caring for a child with IA costed a lot of time and energy with disturbed daily life (16). Moreover, the treatment usually put stress on the family's finances, even causing some families into debt (17). The relationships among family members also be influenced during caregiving, either closer or estranged from each other. Particularly in the case of IA, a disease related to sensitive part of the body, often cause the negative feelings such as embarrassment (12), and such feeling could distorted the family member's attitude toward the affected children (18, 19), thus cause possible deviation.

But from positive prospective, the connection between family members could also be strengthened due to the responsibility and attachment toward the care of ill children (20), which could be beneficial to the caregivers. Moreover, some caregivers could also benefit from caregiving experience. Studies reported that during the process of care, caregivers may cherish the present, improve their personal abilities and enhance their perception of various supports (21).

The different of reactions of caregivers not only influence their personal status, but also have an effect on the quality of caregiving, further influence the prognosis of the children with IA (22, 23). Caregivers of children with IA as independent individual, have their own patterns of reaction. To identify the caregiver's reaction patterns is critical to adaptation and effective coping (24, 25), and help medical staff to understand the caregiver's experience and further design targeted interventions.

But, study related to the different reactions of caregivers of children with IA was lacking. Most studies focused on the care burden of caregivers (26), the complexity of caregiver reaction was overlooked. Thus, in this study, we aimed to identify different patterns of caregiver's reaction by categorized individuals according to their Caregivers Reaction Assessments (CRA) scores, and explore the factors associated with different reaction patterns.

## Methods

### Data Source

The study design, enrollment criteria and hospitals involved were published before (27). The whole project was aiming to explore the caregiving reaction of caregivers of children with imperforate anus using both quantitative and qualitative methods. In this paper, the classification of caregivers reaction and associated factors were identified.

### Measurements

#### Latent Variable: Caregiver Reaction

The caregiver's reaction were collected by Chinese Version of CRA (28), which was developed by Given, and widely used to assess caregiver's burden and benefit (15). The scale comprises 24 items, and each item is rated on a 5-point Likert scale from 1 (strongly disagree) to 5 (strongly agree). The scale consisted five dimensions-impact on health, impact on finances, lack of family support, impact on schedule and caregiver's esteem. The former four dimensions referred the burden of caregivers, and higher score suggested heavier care burden; the last one was the measurement of positive reaction of caregiving, and the higher scores indicated the higher perception of benefit (28). In this study, the Cronbach's Alpha was 0.772 for the scale, and ranged from 0.649 to 0.822 for each dimension.

#### Independent Variable

##### Stigma

The stigma was measured using Chinese version of Parent Stigma Scale (27). It is a one-dimension scale with five items, and each item was rated from 1 (strongly disagree) to 5 (strongly agree), and high scores indicate strong feeling of stigma (29). The Cronbach's Alpha was 0.883 in this study, and the total scores was used as an independent variable.

##### Social Support

In this study, social support was measured by Social Support Scale developed by Xiao (30), the 10-item scale comprising three dimensions as subjective support, objective support and social support utilization. Four items in this scale were rated by four point (1–4), and other items were calculated by the number of chosen option. The high scores the caregivers got, the higher social support level they had. In this study, the Cronbach's Alpha was 0.814 and the total score was used as an independent factor.

##### Perception of Uncertainty

The Chinese revised version of Parent's Perception of Uncertainty (PPUS) was used to measure caregiver's uncertainty feelings in this study (31). The 28-item scale included four dimension as ambiguity, lack of clarity, lack of information, unpredictability. Each item was ranked by the Likert 5-point scale, from 1 (strongly disagree) to 5 (strongly agree), and higher scores indicated higher perception of uncertainty (31). The Cronbach's Alpha was 0.844 in this study. The total scores of the scale was used as an independent variable.

##### Demographic and clinical information

The demographic information and clinical data were collected by self-designed questionnaire, for detailed please to found our previous study (27).

### Data Collection Procedure

The data collection procedure was available in the previous publication (27).

### Data Analysis

Continuous data was expressed by mean and standard deviation. The differences between groups were analyzed using Student's *t* test. Categorial data were expressed by frequencies and proportions and analyzed using Chi-square test. The α would be corrected ($\alpha^{{\;}^{\prime }}=\frac{\alpha }{N}\;N=\frac{n\left(n-1\right)}{2},\;$n: number of groups) (32) when multiple comparisons presented. Dependent variables would be enrolled in logistics regression analysis when *P* < 0.25 (33), clinical significant were also considered before analysis. The analysis was performed in SPSS version 21.0 (IBM Corporation, Armonk, NY, USA).

Patterns of caregiver's reaction were determined using latent profile analysis (LPA). LPA aims to identify clusters of individuals based on a series of continuous variables (34). In this study, the five dimensions of CRA were used as basis for the analysis of latent profile that generate the different reaction pattern. The two-step strategy was used to analyze the data. Firstly, to identify the best-fit model with an initial assumption of one class, and increase the number of classes in sequence, in order to find the best-fitted model. The model fit was also assessed, and typically a relative high Entropy, and a low Bayesian information criterion (BIC) or adjusted BIC (aBIC) indicated good classification (35). The Lo-Mendell-Rubin (LMR) (*P*) and bootstrapped likelihood ratio test (BLRT) (*P*) were used to help determine best class solution, and *P* < 0.05 indicate that the number (n) of classification is better than n-1 (35). The optimal choice of class solution was also evaluated by the interpretability and clinical judgement. This step was conducted using Mplus version 7.1 (Muthén & Muthén, Los Angeles, CA). Secondly, the outcome of selected classification was used as independent variable, and logistics regression analysis was used to determine the associated factors of different classes (conducted by SPSS version 21.0). *P* < 0.05 was considered statistically significant.

### Ethical Approval

The study was approved by the ethical review committee (2018-IRB-081). Informed consents were obtained from each participant which referred that they had entirely understood the study.

## Results

The demographic and clinical information of sample, and the results of stigma and social support were available in previous publication (27). The scores of five dimensions of CRA and PPUS were presented in Table 1.

**Table 1:** Measurement of CRA and PPUS.

	**Total score (range)**	**Item average score (range)**
**Dimensions of** **CRA**		
Health	10.74 ± 2.63 (4.00–18.00)	2.69 ± 0.66 (1.00–4.50)
Finance	8.77 ± 2.77 (3.00–15.00)	2.92 ± 0.92 (1.00–5.00)
Lack of Family Support	11.22 ± 3.19 (5.00–22.00)	2.24 ± 0.64 (1.00–4.40)
Schedule	18.00 ± 3.60 (5.00-−25.00)	3.60 ± 0.71 (1.00–5.00)
Caregiver's Esteem	29.01± 3.20 (20.00–35.00)	4.14 ± 0.46 (2.86–5.00)
Perception of Uncertainty	72.60 ± 14.28 (29.00–102.00)	2.59 ± 0.51 (1.04–3.64)

*CRA, Caregivers Reaction Assessment; PPUS, Parent's Perception of Uncertainty*.

### Classification of Caregiver's Reaction Patterns

**Figure 1:**
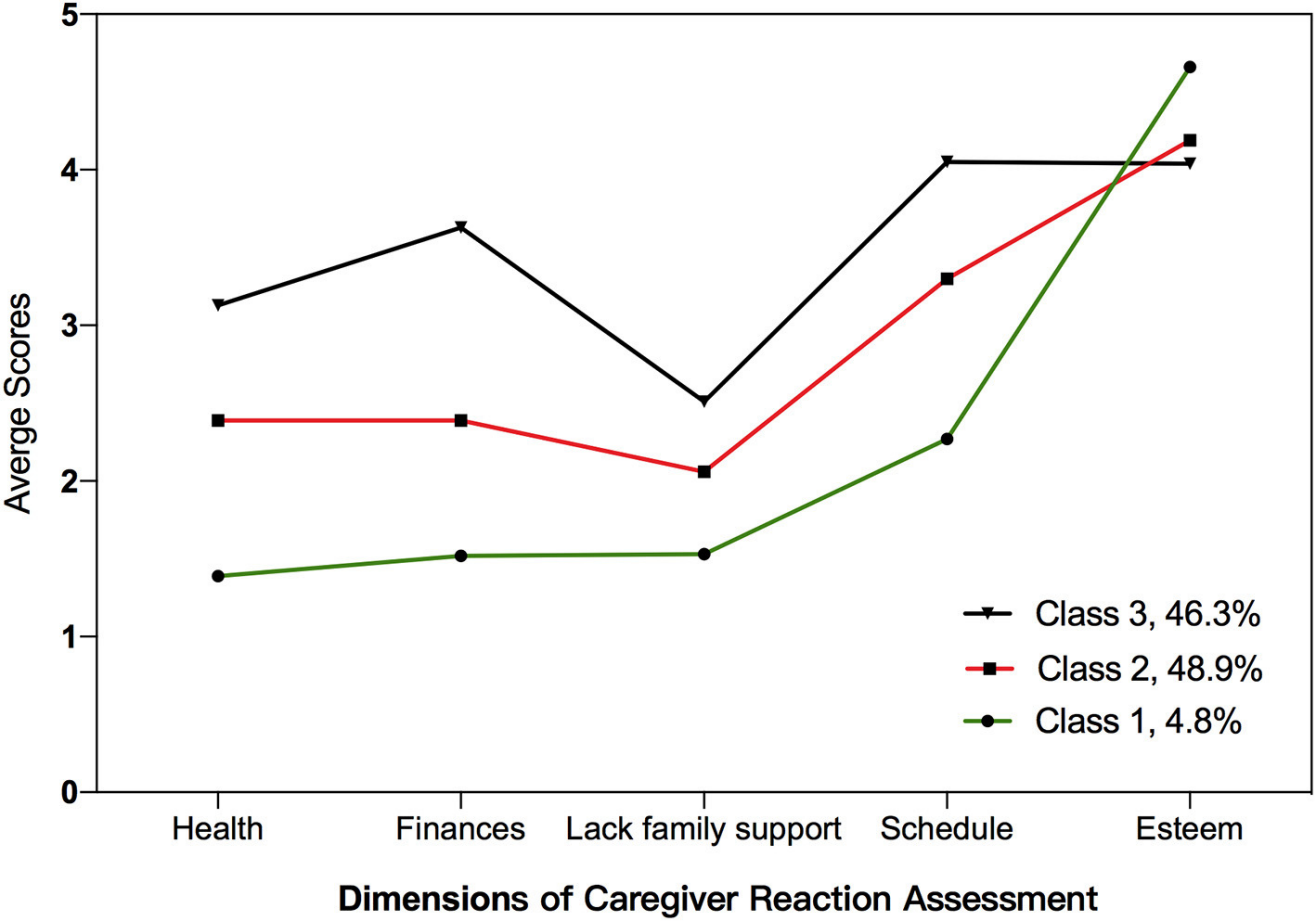
Dimension of caregiver reaction assessment.

The analysis of patterns of caregiver reaction was done based on the results of five dimensions of CRA. The Entropy and BIC indicate that four-class model fitted well, while the LMR test showed that, the four-class model was no better than three-class model. The advantage of four-class model was trivial compared with three-class model, and the Entropy of three-class model is 0.781, indicating the three class model also had high classification certainty. According to our clinical judgement, the three-class model was found to fit a meaningful clinical interpretation, and it was chosen as the final classification model. The statistics of model fit indices were depicted in Table 2. Figure 1 showed the results of three-class model, giving the characteristics of the five dimensions, the former four dimension could defined as caregiving burden, and the last dimension could view as sense of benefit. When comparing the three classes, the label of 3 classes could be defined as low burden and high benefit (Class 1, 4.8%), moderate burden and benefit (Class 2, 48.9%) and high burden and low benefit (Class 3, 46.3%). In Class 1, esteem got the highest scores, followed by schedule, lack of family support, finances and health. In Class 2, esteem got the highest scores as well, followed by schedule, finance, health and lack of family support. In Class 3, schedule got the highest scores, followed by esteem, finances, health and lack of family support (Table 3).

**Table 2:** Model fit indices.

**Classes**	**k**	**Log (L)**	**AIC**	**aBIC**	**Entropy**	**LMR test**	**BLR test**
1	10	−1147.088	2314.176	2316.820	—	—	—
2	16	−1064.555	2161.109	2165.339	0.708	0.0002	<0.001
3	22	−1043.691	2131.382	2137.198	0.781	0.0194	<0.001
4	28	−1033.495	2122.990	2130.393	0.815	0.0892	<0.001

*AIC, Akaike Information Criterion; BIC, Bayesian Information Criterion; LMR, Lo-Mendell-Rubin; BLR, bootstrapped likelihood ratio*.

**Table 3:** Descriptive statistics for five dimensions of CRA in three classes.

	**Class 1**	**Class 2**	**Class 3**
Health	1.39 ± 0.38	2.39 ± 0.44	3.13 ± 0.50
Finances	1.52 ± 0.55	2.39 ± 0.63	3.63 ± 0.62
Lack of family support	1.53 ± 0.39	2.06 ± 0.52	2.51 ± 0.65
Schedule	2.27 ± 0.76	3.30 ± 0.57	4.05 ± 0.46
Esteem	4.66 ± 0.27	4.19 ± 0.42	4.04 ± 0.47

*Class 1, low burden and high benefit, 4.8%; Class 2, moderate burden and benefit, 48.9%; Class 3, high burden and low benefit, 46.3%*.

### Associated Factors of Different Reaction Patterns

**Figure 2:**
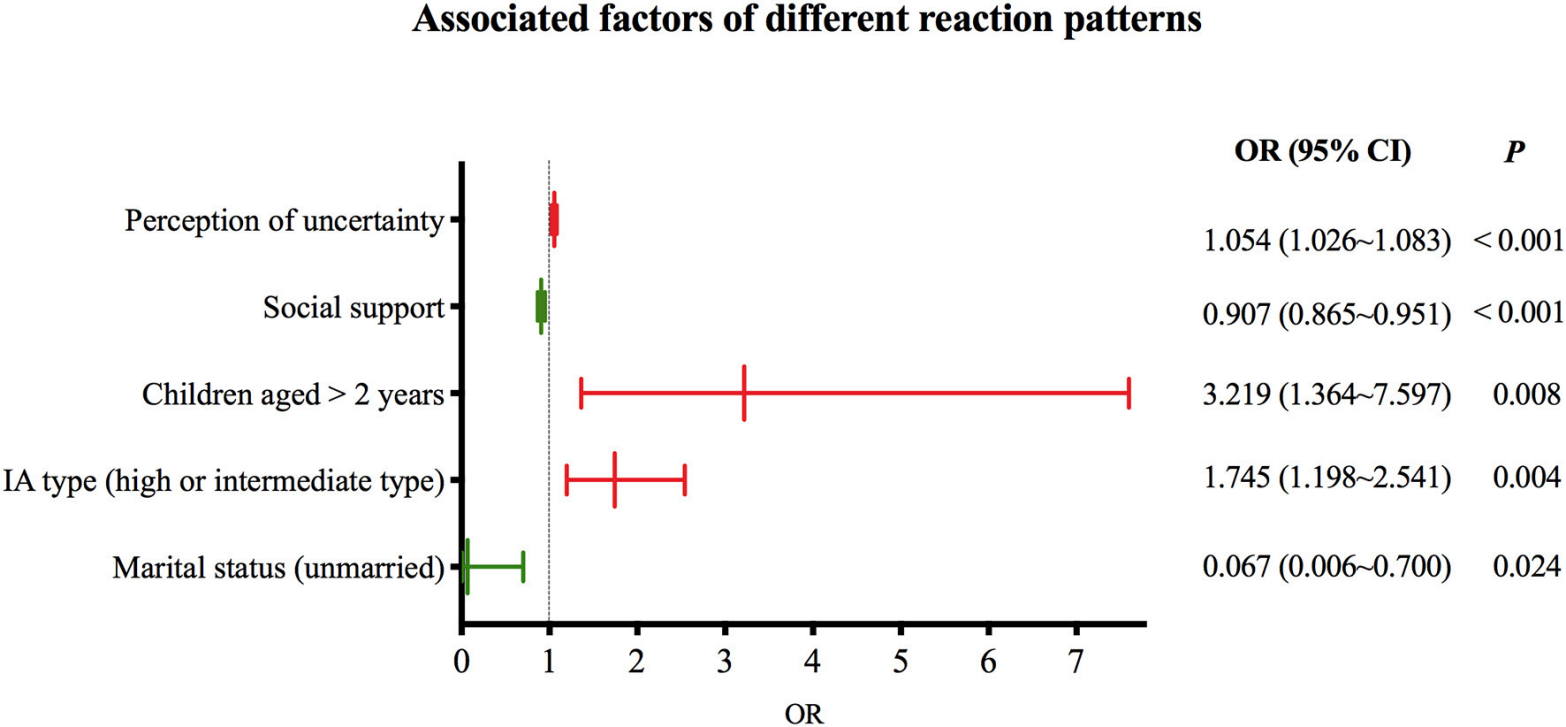
Associated factors of different reaction patterns.

In the logistics regression analysis, due to the small number of population in Class 1, the Class 1 was combined with Class 2 as one category, defined as low burden and high benefit group, the Class 3 remained as high burden and low benefit group. The stepwise method were also used to determine the independent associated factors derived from univariate analysis (Table 4). The results showed that marital status, IA type, children's age, social support and perception of uncertainty were factors that associated with different reaction patterns (Figure 2).

**Table 4:** Univariate analysis of different reaction patterns.

**Item**	**Class 1**	**Class 2**	***χ2/t/z***	***P-*value**
**Caregiver**				
**Age (years)**			1.079	0.583
<30	55	45		
30–40	58	48		
>40	10	13		
**Gender**			0.125	0.724
Male	21	20		
Female	102	86		
**Marital status**			— — –	0.127
Married	117	105		
Other	6	1		
**Education level**			2.816	0.421
Primary school or below	7	10		
Junior high school	33	35		
High school	25	20		
University/college or above	58	41		
**Occupation**			16.148	<0.01
Part-time job	11	21		
Full-time job	59	25		
Unemployed	53	60		
**Relationship with patient**			0.581	0.446
Mother	98	80		
Father and other	25	26		
**Residence**			7.679	0.022
City	42	19		
Suburban	37	39		
Countryside	44	48		
**Household structure**			0.159	0.690
Extended family	82	68		
Nuclear family	41	38		
**Religion**			0.106	0.745
Yes	37	34		
No	86	72		
**Children**				
Age (years)			5.123	0.024
≤ 2	108	81		
>2	15	25		
**Gender**			0.588	0.443
Male	80	74		
Female	43	32		
**IA typ**e			19.335	<0.01
Low	82	42		
Intermediate	20	21		
High	21	43		
**Time since diagnosis (years)**			3.744	0.053
<1	76	52		
≥1	47	54		
**Medical insurance**			4.764	0.029
Yes	100	73		
No	23	33		
**Concealment**			0.023	0.879
Yes	87	74		
No	36	32		
Stigma	13.68 ± 4.68	16.48 ± 4.55	−4.583	<0.01
Social support	43.40 ± 7.83	36.77 ± 7.52	6.520	<0.01
Perception of uncertainty	67.37 ± 13.28	78.63 ± 12.47	−6.615	<0.01

*Class 1, low burden and high benefit/moderate burden and benefit; Class 2, high burden and low benefit*.

## Discussion

This study aimed to identify different reaction patterns of caregivers of children with IA during follow-ups, and explore associated factors that determined different types of reaction. The results showed that three distinguishable patterns were identified as Class 1 (low burden, high benefit), Class 2 (mediate burden and benefit) and Class 3 (high burden, low benefit). About 46% of the population experienced high burden and low benefit compared to others. Got married, children aged > 2 years and diagnosed with higher type of IA, less social support and perception of uncertainty may negatively affect the caregiver's reaction. To better understand the different reaction patterns and the influencing factors, could not only instruct the medical staff to provide caregivers with specific interventions, but also help the children and their families to get better transition from initial years into the adulthood period.

There was some similarity among the three classes of reaction pattern. Each pattern shared a relative high esteem score compared to other dimensions, which referred that the caregivers of children with imperforate anus could experience much positive feeling during care. When comparing different classes, the population could be mainly divided into two groups as low burden and high benefit group and high burden and low benefit group, because in Class 1 and Class 2, the esteem all top the five dimensions of caregiver reaction assessment, which presented that the caregivers in these two groups perceived more positive feelings than negative ones, and these population could accounted for 53.7%. Thus, it is important for us to notice the positive feelings that caregivers experienced. The positive reaction from caregiving, could not only impact the caregiver's motives to provide high quality care for the children with IA, but also could reduce the sense of guilty of the children when they grow-up with self-conscious.

The positive feelings, sometimes called benefit finding, was generated from the process of disease coping (21). Benefit finding is a common phenomenon to see in the caregivers of children with chronic illness (14). Because caregivers usually have close relationships with the ill children, they will attach themselves with great possibility to give the children best care, which could also foster the caregivers to find positive meaning during care (24). Therefore, in the long-term of caregiving, it was important for the clinical medical staff to guide the caregivers to treat the illness objectively, especially share the benefit findings with their children, and lead their attitude toward positive side. Interventions like writing disclosure, encouraging the caregivers to spend 20 min a day to record the positive feeling during caregiving (36), could be taught to the caregivers to enhance sense of benefit, which could finally help reduce their care burden, and increase the care quality for children with IA. These could also benefit the children from learning the experiences in the procession, which could facilitate the transition into adulthood.

As for the care burden, impact on schedule in three patterns all top the dimensions of negative reaction, indicated that caregivers suffer most from the disturbed daily arrangement. That is the treatment of IA is a long period, during which, the caregivers need to adjust their daily schedule and decrease their social activities to spend more time to care the children (16). To reduce the negative impact on schedule, on one hand, the caregivers could learn more nursing skills to improve their efficiency of care or ask family members at home for help (37). On the other hand, they could empower the children with self-care ability, along with other living skills in their growth. With the self-care skills, the children with IA could better deal with the chronic disease and make a smooth transition into adulthood.

To our surprise, the married caregivers had more possibility to experience negative pattern of reaction, which referred that they usually burden negative effects and benefit less compared to unmarried caregivers. The possible explanation may be that, the unmarried caregivers have less pressure from spouses (38), because when someone take the responsibility of care, others will take for grant that the children should be cared well. If there is something wrong with the children, the caregivers will be blamed as careless. Especially in the case of mother, who accounted the majority (77.7% in this study) of the caregivers, even the incidence of IA could be attributed as their fault, and this phenomenon was evident in the results of qualitative part of our study (17). In clinical practice, we should view the children's family as a unit (26), not only caregivers need to know the knowledge and nursing skills for children's, but also other family members. This could help the families of children with IA to care the children together, or at least, enhancing their understanding of the hard caring tasks may help improve their attitude toward caregivers, thus modifying the caregiver's reaction during caring.

In this study, young children aged > 2 years was found to be an factor that caused caregivers react negatively toward caregiving. That is, although most treatment were performed in the initial years, frequent follow-ups were still needed when children grown up with self-awareness (11). Also, the unexpected complications such as soiling and constipation (11) may play a part in shaping their negative reaction pattern. So special care should be paid for the caregivers of children aged > 2 years. Useful materials should be provided to assist the caregivers cope with the chronic situation and regular follow-ups through telephone may be help.

The IA type was also important in determining different reaction patterns. Caregivers of children diagnosed with intermediate or high type of IA usually experience more burden and less benefit from caregiving. That is, compared with low type IA, the other two types needed more complex treatment and longer time of recovery (39). For example, after colostomy operation for intermediate or high type IA, the caregiver need to nurse the colostomy careful to avoid skin injuries and other complications. Moreover, the higher the malformation is, the relative poor prognosis the children will be (39). As the children of intermediate or high type of IA usually have poor-developed interior sphincters, as well as the later reaction of nerve reflexes (40). This could negatively affect the quality of life of children with IA, and the caregivers reaction will be negative correspondingly. To promote caregiver's understanding of characteristics of different IA types may help them to accept the reality they need to face after discharging from hospital, and adjust their anticipation for the children's prognosis, further modify their reaction when caring. With reasonable anticipation, the caregivers could better help the children to accept the possible prognosis they may encounter during their growth into adulthood.

In this study, higher level of social support and less perception of uncertainty could positively affect caregiver's reaction. It was consistent with other studie's results that social support and reduction of uncertainty has the potential to reduce caregiver's burden. Therefore, medical staff should offer sufficient information before they discharging, which could help to alleviate the caregiver's uncertainty feelings during care.

Normally, the perception of uncertainty could cause anxiety, increase caregiver's burden and reduce their sense of benefit (41). However, the effect of uncertainty could be both beneficial or harmful depending on one's view (42). Because uncertainty could also be viewed as possibility of getting a better result (43). Thus, besides providing detailed explanations for the caregiver's concerns, it is also crucial to guide them think positively about the prognosis when something unexpected happened. This could aid in the improvement of caregiver reaction, as well as inspire the caregivers to cultivate the optimistic character of children with IA.

Social support is an important protective factor of improving individual's well-being (44), thus it also should be used to elevate caregiver's sense of benefit during care. Moreover, peer support is also matters (45) other than the support from family members and medical staff. To invite former patients who had successful transition experiences to adulthood, especially those with positive attitude may help the caregivers to sense more benefit in caring and enhance their perception of social support (30). These success examples could also encourage the children to have a better transition toward adulthood. The results of social support measurement in this study showed that, the objective social support that caregivers got was the highest among three dimensions, however, their subjective social support, especially social support utilization was relative low, which indicated that the caregivers of children with IA may not make full use of their social support. Therefore, medical staff could encourage the caregivers to keep a gratitude dairy to appreciate the help they have received (46), thus increase their perception of social support and make full use of it. This could help them to reduce care burden and promote their sense of benefit.

## Conclusion

The caregivers of children with IA could experience high level of benefit during care, but also take a lot of burden like disturbed schedule. According to their reaction pattern, the caregivers could be mainly divided into two groups, namely low burden, high benefit group and high burden, low benefit group. Unmarried caregivers have greater possibility to experience positive reaction pattern. However, when children > 2 years and diagnosed with intermediate or high type of IA, the caregivers may have negative reaction pattern. But, increasing caregiver's social support level, exploring more social support resources, as well as enhancing their perception and utilization of the support they have received, could help them modify their reaction. Moreover, guiding the caregivers to view positively about the fact they faced, and take the uncertainty as possibility may also help them to improve their reaction patterns.

## Limitation

There were several limitations in this study. Firstly, the measurement of benefit finding of caregiving was not enough to illustrate the situation. Only one dimension of CRA was used may not stand for the benefit finding of caregivers, and we performed a qualitative research as complement to study more about the benefit finding of this population. Quantitative exploration of benefit finding of caregivers are needed in future studies. Secondly, the nature of cross-sectional study could not help us to determine the direction of correlation, for example, the different reaction patterns may also affect caregiver's perception of uncertainty, and we could not capture the changes of reaction patterns with time as well. In addition, although three categories were identified in LPA, but due to the small number of the sample size, limited members distributed in Class 1, which combined with Class 2 as one category in the logistics analysis. Larger sample size are needed in future study to explore the specific influencing factors associated with distinctive and refined reaction patterns. Lastly, this study did not include the clinical outcome of the children with IA, such as complications, hospitalization time, follow-up frequency and so on. Further explorations could be performed to see the relationships between caregiver's reaction patterns and the clinical outcome of their children, which could provide more evidence of nursing interventions.

## Data Availability Statement

The raw data supporting the conclusions of this article will be made available by the authors, without undue reservation.

## Ethics Statement

The studies involving human participants were reviewed and approved by Ethics Committee Board of Children's Hospital of Zhejiang University, School of Medicine. The patients/participants provided their written informed consent to participate in this study.

## Author Contributions

HX made the contribution to conception and design. DW, KL, JT, YJ, and WG made substantial contribution to design, acquisition of data, analysis, and interpretation of data. DW draft the manuscript. XC and FL revising it critically for important intellectual content. Each author gave the final approval to the submitted version.

## Conflict of Interest

The authors declare that the research was conducted in the absence of any commercial or financial relationships that could be construed as a potential conflict of interest.

## Publisher's Note

All claims expressed in this article are solely those of the authors and do not necessarily represent those of their affiliated organizations, or those of the publisher, the editors and the reviewers. Any product that may be evaluated in this article, or claim that may be made by its manufacturer, is not guaranteed or endorsed by the publisher.
